# Coronary Sinus Reduction for REDUCER-I Patients With Refractory Angina and Angiographically Nonobstructive Coronary Artery Disease

**DOI:** 10.1016/j.jacadv.2026.102686

**Published:** 2026-03-20

**Authors:** Ranil de Silva, Tim P. van de Hoef, Jan-Peter van Kuijk, Jonathan Byrne, Matteo Montorfano, Eva Buschmann, Shmuel Banai, Jennifer Luyapan, Nick E.J. West, Stefan Verheye

**Affiliations:** a Royal Brompton and Harefield Hospitals, Part of Guy’s and St Thomas’s NHS Trust and National Heart and Lung Institute, Imperial College, London, United Kingdom; b University Medical Center Utrecht, Utrecht, The Netherlands; c St. Antonius Ziekenhuis Hospital, Nieuwegein, The Netherlands; d King's College Hospital, London, United Kingdom; e School of Medicine, Vita-Salute San Raffaele University, Milan, Italy; f Interventional Cardiology Unit IRCCS San Raffaele Scientific Institute, Milan, Italy; g Medical University Graz, Graz, Austria; h Tel Aviv Medical Center and Tel Aviv University School of Medicine, Tel Aviv, Israel; i Shockwave Medical Inc, Santa Clara, California, USA; j Cardiovascular Center, ZAS Middelheim Hospital, Antwerp, Belgium

**Keywords:** coronary artery disease, coronary sinus Reducer, non-obstructive, refractory angina

## Abstract

**Background:**

Patients with angiographically nonobstructive coronary artery disease (CAD) (AngioNOCAD), including those with previous successful revascularization, often experience refractory angina (RA).

**Objectives:**

The objective of the study was to assess the efficacy of coronary sinus (CS) Reducer for the treatment of patients with AngioNOCAD and no further revascularization options.

**Methods:**

The REDUCER-I registry is a nonrandomized, real-world observational study of patients with RA, objective evidence of ischemia on noninvasive stress testing, and with no further revascularization options. AngioNOCAD was defined as < 70% stenosis by visual adjudication in all major epicardial coronary arteries at time of enrollment. 12-month outcomes included Canadian Cardiovascular Society class, Seattle Angina Questionnaires (SAQ), and major adverse events.

**Results:**

Of the 371 patients who received a CS Reducer implant, 306 reported baseline CAD type, 61/306 (19.9%) had AngioNOCAD, and 245/306 (80.1%) had obstructive CAD. Both groups had similar baseline characteristics although the AngioNOCAD patients were younger (65.8 ± 11.3 years vs 70.2 ± 8.7 years), had less prior coronary artery bypass grafting (44.3% vs 79.6%), and more prior percutaneous coronary intervention (44.3% vs 71.8%) than patients with obstructive CAD. Twelve months after CS Reducer implantation, 60.3% of the AngioNOCAD cohort and 71.7% of the obstructive CAD cohort had ≥1 Canadian Cardiovascular Society class improvement (*P* = 0.11). Twelve-month MACE rates were similar (AngioNOCAD 7.0%, obstructive CAD 8.0%), and both groups had significant improvements in SAQ angina frequency, quality of life, and mean SAQ summary score (*P* < 0.01).

**Conclusions:**

In the REDUCER-I registry, patients with RA and AngioNOCAD had significant improvements in their symptoms and quality of life suggesting that CS Reducer implantation may be an effective therapy in these patients.

Angina affects over 100 million people worldwide with approximately half of cases associated with angiographically nonobstructive coronary artery disease (CAD) (AngioNOCAD).^1^ A contemporary definition for refractory angina (RA) has been proposed, “a chronic condition of at least 6 months duration caused by myocardial ischemia triggered by obstructive CAD and/or other mechanisms, and is characterized by the persistence of angina or angina-equivalent symptoms despite maximally-tolerated stratified antiischemic therapy and achievable indicated revascularization”, to better reflect the complexity of ischemic mechanisms underlying RA.^2^ Importantly, this definition includes AngioNOCAD patients who may have a history of obstructive CAD but continue to experience angina symptoms even after successful revascularization procedures. These AngioNOCAD patients should be considered distinct from patients conventionally defined as having angina with nonobstructive coronary arteries (ANOCA), in whom angina symptoms may result from multiple mechanisms including coronary microvascular dysfunction (CMD) and abnormal coronary vasomotion demonstrated by coronary function testing.^1^^,^^3^

Improvement in symptoms and quality of life are key treatment objectives for patients with RA.^2^ In patients with RA and obstructive CAD, randomized sham-controlled clinical trials have demonstrated significant reductions in physician and patient-adjudicated angina^4^^,^^5^ after coronary sinus (CS) Reducer (Shockwave Medical Inc) implantation. For patients with nonobstructive CAD, including those with previous successful percutaneous or surgical revascularization,^2^^,^^6^ possible causes of ischemia resulting in angina include endothelial or CMD, microvascular or epicardial coronary spasm, or myocardial bridging.^2^^,^^7^ Although stratified pharmacological treatment of nonobstructive CAD based on the underlying mechanistic endotype can improve symptoms,^8^, ^9^, ^10^ many patients remain symptomatic with RA. CS Reducer may be beneficial in some of these patients, with several preliminary reports indicating that narrowing the CS improves microcirculatory function by decreasing microvascular resistance and increasing coronary flow reserve.^11^^,^^12^ In an open-label study of patients with angina and unobstructed coronary arteries, CS Reducer implantation resulted in significant improvements in microvascular function, better quality of life indices, and improved angina symptoms.^13^

The REDUCER-I study (NCT02710435) evaluated the real-world use of the CS Reducer in patients with RA and a history of CAD despite revascularization and optimized medical therapy.^14^^,^^15^ We report an analysis of the effect of CS Reducer implantation in a subgroup of patients with AngioNOCAD.

## Methods

The design of the REDUCER-I study has been detailed previously.^14^^,^^15^ Briefly, this was an international, multicenter nonrandomized study. All enrolled patients (n = 400) had RA, demonstrable myocardial ischemia despite optimized medical therapy, and no further revascularization options. All patients had a history of CAD and were in Canadian Cardiovascular Society (CCS) class II-IV angina. Of the 400 enrolled patients, 371 were implanted with a CS Reducer. For this subanalysis, patients were defined as having AngioNOCAD if they had <70% stenosis by visual adjudication in any major epicardial coronary artery or epicardial bypass grafts. The <70% threshold is consistent with earlier publications^16^, ^17^, ^18^ although visual estimation of coronary stenosis can have limitations including the potential overestimation of lesion severity.^19^ Importantly, the degree of stenosis was assessed at time of enrollment and patients could have had patent stents or grafts due to prior successful percutaneous or surgical revascularization procedures provided all other enrollment criteria were met. Of the 371 patients implanted with a CS Reducer, dichotomization of patients into the obstructive or AngioNOCAD groups at baseline was possible in 306 patients.

Informed consent was obtained from each patient and all sites were required to follow local legal and regulatory requirements for Ethics Committee and Institutional Review Board approvals. The study was conducted in accordance with the Declaration of Helsinki guidelines and Good Clinical Practices. An independent Clinical Events Committee was responsible for adjudicating the protocol-defined adverse events. Endpoints evaluated at 12 months included change in CCS class compared with baseline and major adverse cardiac events (MACE), a composite of cardiac death, major stroke, and myocardial infarction (MI). Angina was assessed using CCS classification and the Seattle Angina Questionnaire (SAQ).^20^

### Statistics

Continuous variables are shown as mean ± SD with comparisons performed using a Student t-test or Wilcoxon rank-sum test. Categorical variables are expressed as percentages and compared with a chi-square test, Fisher exact test, or McNemar’s chi-square test. Kaplan-Meier time to event estimates were used for analysis of freedom from MACE and paired *t*-tests were used to analyze change from baseline in SAQ domains. A multivariate logistic regression analysis was performed to assess the association of obstructive CAD vs AngioNOCAD on having ≥1 CCS class improvement at 12 months. Prespecified covariates of age, sex, diabetes, baseline CCS, and baseline SAQ were selected based on historical clinical relevance and to control for observed differences at baseline. Analyses were conducted using a complete case approach, any subjects with missing data at 12 months were excluded from the model, and no imputation was performed. ORs, 95% CIs, and *P* values for each variable in the regression analysis are presented in a forest plot. All analyses were performed using SAS 9.4 (SAS Institute Inc).

## Results

**Figure 1:**
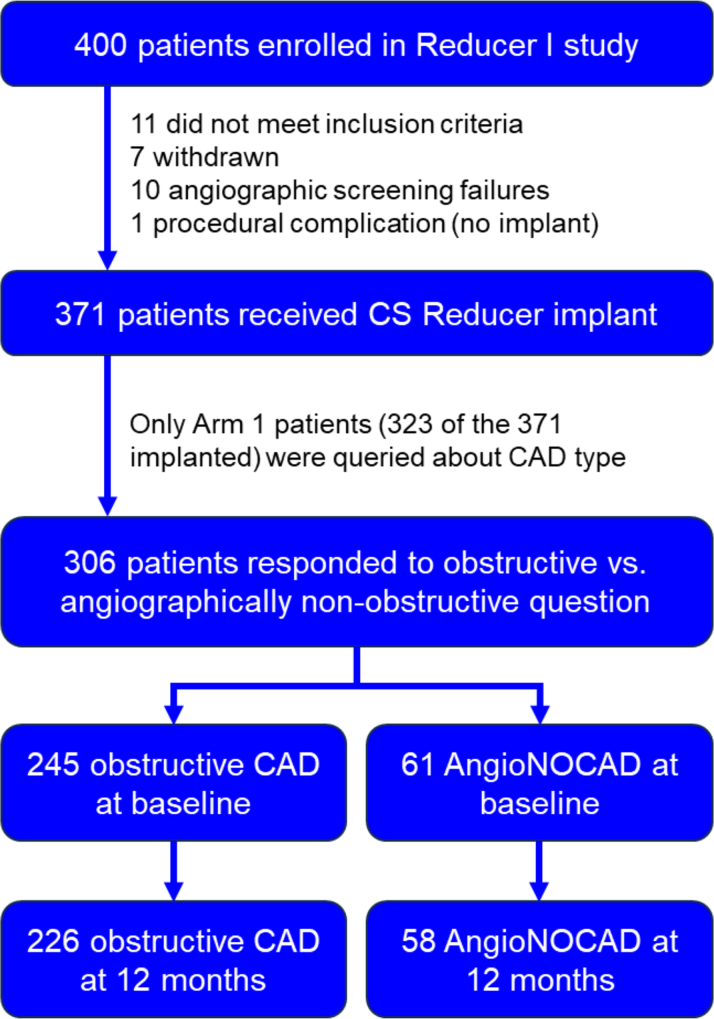
**CONSORT Diagram of Obstructive Coronary Artery Disease and Angiographically Nonobstructive Coronary Artery Disease Patients** CONSORT diagram showing of the 400 patients originally enrolled in REDUCER-I, 306 were classified as having obstructive CAD (n = 245) or AngioNOCAD (n = 61) and their follow-up through 1 year. CAD = coronary artery disease; AngioNOCAD = angiographically nonobstructive CAD; CS = coronary sinus.

In the 306 patients with available data on coronary anatomy, obstructive CAD was present in 245/306 (80.1%) and AngioNOCAD was present in 61/306 (19.9%) at the time of enrollment (Figure 1). Baseline characteristics for the 2 groups are shown in Table 1. Patients with AngioNOCAD were younger than those with obstructive CAD, although there was no difference in sex or baseline CCS class. At baseline, 70.2% (172/245) of obstructive CAD patients and 72.1% (44/61) of AngioNOCAD patients were in CCS classes aIII/IV (*P* = 0.11). In the AngioNOCAD group, 44.3% had prior coronary artery bypass grafting (CABG) and 85.2% had prior percutaneous coronary intervention (PCI), and 55.7% had a history of prior MI. Obstructive CAD patients had similar rates of prior MI although they had significantly higher history of CABG (79.6%, *P* < 0.01) and a trend for less prior PCI (71.8%, *P* = 0.05).

**Table 1:** Baseline Characteristics of Patients With Obstructive CAD and Angiographically Nonobstructive CAD From the REDUCER-I Registry

	Obstructive CAD (n = 245)	AngioNOCAD (n = 61)	*P* Value
Age (y)	70.2 ± 8.7	65.8 ± 11.3	<0.01
Male (%)	79.2%	77.0%	0.85
Prior MI	56.7%	55.7%	1.00
Prior CABG	79.6%	44.3%	<0.01
Prior PCI	71.8%	85.2%	0.05
Hypercholesterolemia	89.8%	82.0%	0.14
Diabetes	49.0%	45.9%	0.77
Hypertension	84.1%	83.6%	1.00
Baseline CCS (%)			0.11
Grade II	29.8%	27.9%	
Grade III	63.7%	57.4%	
Grade IV	6.5%	14.8%	

Values are mean ± SD or %.

AngioNOCAD = angiographically non-obstructive CAD; CABG = coronary artery bypass grafting; CAD = coronary artery disease; CCS = Canadian Cardiovascular Society; MI = myocardial infarction; PCI = percutaneous coronary intervention.

**Figure 2:**
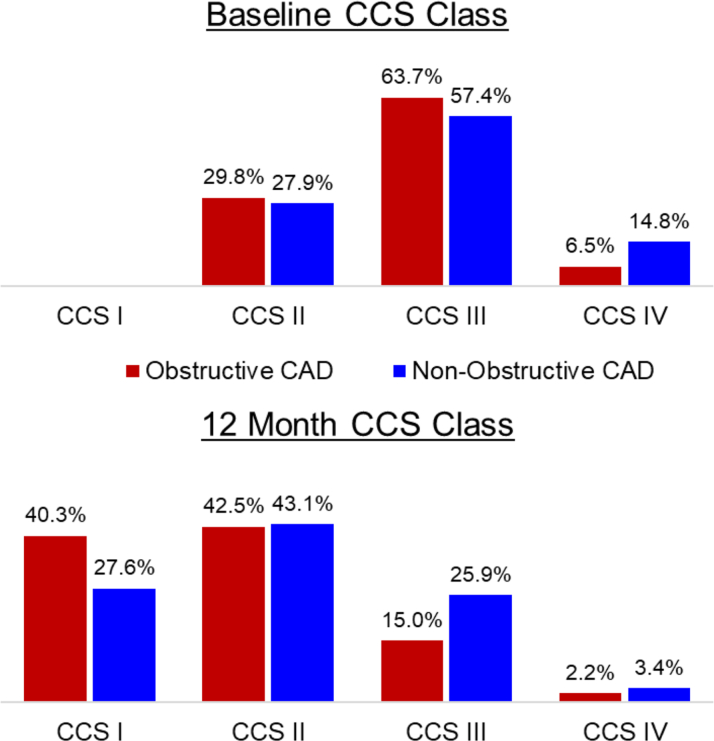
**Change in Canadian Cardiovascular Society Class for Obstructive Coronary Artery Disease and Angiographically Nonobstructive Coronary Artery Disease Patients** Patients with obstructive CAD and angiographically nonobstructive CAD in the REDUCER-I Registry both had significant improvements in CCS class at 12 months as compared to baseline. There were not statistically significant differences between the obstructive CAD and AngioNOCAD groups. CCS = Canadian Cardiovascular Society; other abbreviation as in Figure 1.

**Figure 3:**
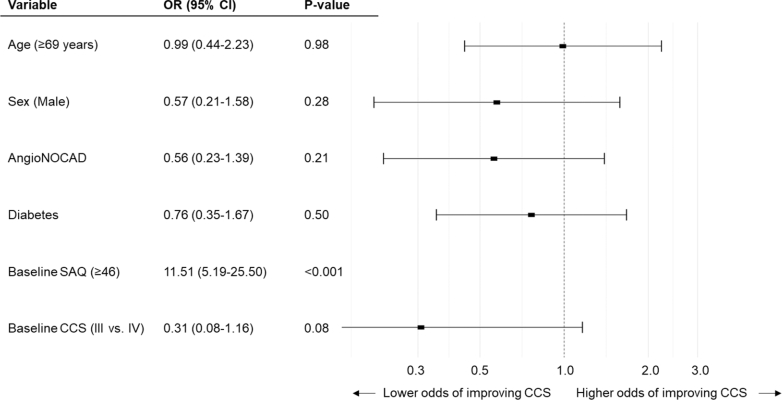
**Predictors of ≥1 Canadian Cardiovascular Society Class Improvement at 12 months** Multivariate logistic regression analyses with stepwise selection were performed to assess predictors of ≥1 CCS class improvement at 12 months. AngioNOCAD was not an independent predictor of ≥1 CCS class improvement at 12 months. SAQ = Seattle Angina Questionnaire; other abbreviation as in Figures 1 and 2.

Twelve months after CS Reducer implantation, both obstructive and AngioNOCAD cohorts experienced improvements in CCS class (Figure 2, additional details in the Supplemental Appendix). There was no difference in the proportion of patients with obstructive CAD (71.7%, 162/226) and AngioNOCAD (60.3%, 35/58) who had ≥1 CCS class improvement at 12 months (*P* = 0.11). 26.1% (59/226) of the obstructive CAD group and 17.2% (10/58) of the AngioNOCAD group had an improvement of ≥2 CCS classes at 12 months, again with no statistically significant difference between the 2 groups (*P* = 0.17). After adjusting for differences in baseline demographics, AngioNOCAD was not an independent predictor of ≥1 CCS class improvement at 12 months (Figure 3).

**Figure 4:**
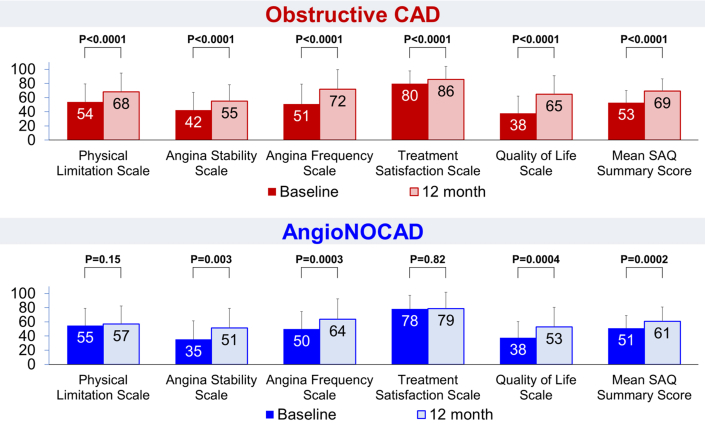
**Seattle Angina Questionnaire Scores for Obstructive Coronary Artery Disease and Angiographically Nonobstructive Coronary Artery Disease Patients** Both obstructive CAD and AngioNOCAD groups had significant improvements in SAQ scores at 12 months, notably in their angina stability, angina frequency, and quality of life scales. Abbreviation as in Figures 1 and 3.

SAQ scores were collected in 245 obstructive and 61 AngioNOCAD patients at baseline and 222 obstructive and 57 AngioNOCAD patients had SAQ data available at their 12-month follow-up. Both obstructive and AngioNOCAD groups had significantly higher SAQ domain scores at 12 months most notably in angina stability, angina frequency, and quality of life scales (Figure 4, additional details in Supplemental Appendix). The mean SAQ summary score improved from 52.9 ± 17.2 at baseline to 69.1 ± 17.2 at 12 months for obstructive CAD patients (*P* < 0.0001). In AngioNOCAD patients, the SAQ summary score increased from 51.0 ± 17.8 at baseline to 60.8 ± 19.9 at 12 months (*P* = 0.0002).

**Figure 5:**
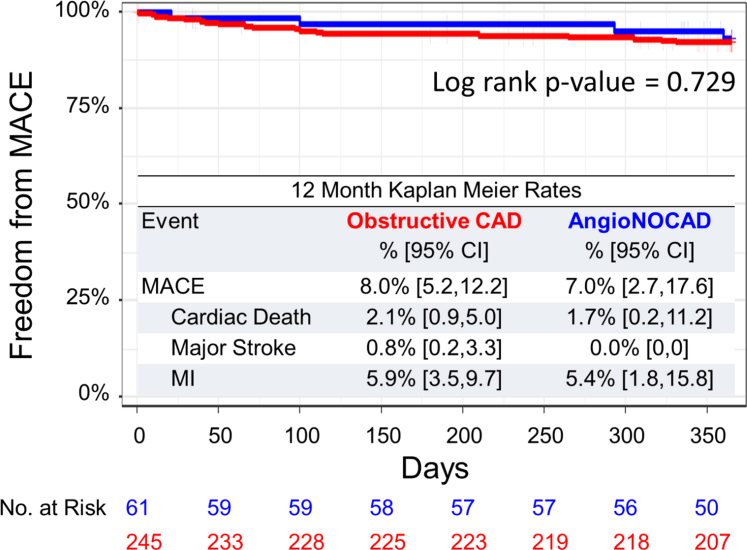
**MACE Kaplan-Meier Estimates for Obstructive Coronary Artery Disease and Angiographically Nonobstructive Coronary Artery Disease Patients** Obstructive CAD and AngioNOCAD patients did not have statistically different rates of MACE through 12 months. MACE = major adverse cardiac events; other abbreviation as in Figure 1.

MACE rates for the obstructive CAD and AngioNOCAD groups are shown in (Figure 5). There were no statistically significant differences in the overall MACE rates between the 2 groups (*P* = 0.73) and MACE was driven by the rates of MI (5.9% for obstructive and 5.4% for nonobstructive).

## Discussion

In this 1 year prespecified subgroup analysis of the obstructive and AngioNOCAD patients from the REDUCER-1 registry, the primary findings were: 1) The population recruited to REDUCER-1 reflects the contemporary and real-world definition of RA including patients with AngioNOCAD or after successful revascularization; 2) at 1 year, both obstructive CAD and AngioNOCAD patients had improved anginal symptoms and quality of life after CS Reducer implantation; 3) the reduction in angina frequency, improvement in CCS class, and SAQ summary scores were similar to the results reported in randomized controlled trials.^4^^,^^5^

The mechanism by which CS Reducer implantation relieves angina have been attributed to redistribution of myocardial blood flow toward ischemic segments as an effect of Reducer implantation according to studies with quantitative perfusion assessments.^5^^,^^21^, ^22^, ^23^ The ORBITA COSMIC trial demonstrated redistribution of blood flow particularly toward subendocardial layers in visually adjudicated ischemic segments,^5^ consistent with preclinical studies.^24^ A recent modeling analysis suggests that CS Reducer implantation in cases of moderate coronary stenosis can increase capillary transit time to aid tissue oxygenation.^25^ Although the current study did not include imaging assessment of changes in myocardial blood flow, it is plausible that these previously described mechanisms may have resulted in the symptomatic benefits seen in the AngioNOCAD group.

In the AngioNOCAD group, there was a high rate of previous PCI and CABG. Persistence of angina after revascularization is common and can occur despite successful, durable, and complete epicardial coronary revascularization.^26^ Residual myocardial ischemia could be due to diffuse epicardial coronary disease, CMD, epicardial/microvascular spasm, or myocardial bridging.^2^^,^^6^ An appropriate clinical evaluation of the stratified therapy for these patients has recently been proposed.^2^ However, pharmacologic treatment of persistent angina after revascularization in the context of AngioNOCAD remains a clinical challenge,^27^^,^^28^ and randomized double blind clinical trials of medical therapy stratified according to mechanisms of ischemia in patients with persistent angina and nonobstructive CAD after revascularization are not currently available.

The results from the REDUCER-I registry suggest that the CS Reducer may be an effective treatment for appropriately selected patients with persistent angina and AngioNOCAD. This intervention may be particularly beneficial for those patients in whom CMD is the predominant mechanism of ischemia due to the potential redistribution of myocardial blood flow after CS Reducer implantation.^29^ Other potential nonpharmacologic treatment options could include enhanced external counter pulsation, extracorporeal shockwave therapy, biologicals, and neuromodulation but many of these remain investigational.^2^ CS Reducer implantation can be performed with high procedural success and has met safety and effectiveness endpoints in a randomized sham-controlled trial.^30^ These safety results have been corroborated in this large real-world cohort in patients with both obstructive CAD and AngioNOCAD. The symptomatic improvements observed in patients with AngioNOCAD in the current report are also consistent with those observed in the nonobstructive CAD subgroup of the RESOURCE registry^31^ and ANOCA patients treated with the CS Reducer.^13^

In the AngioNOCAD group, the MACE rate after CS Reducer implantation is low and similar to other studies in patients undergoing revascularization by PCI.^32^^,^^33^ The rate of MI in this study is numerically higher than has been reported in prior studies,^16^, ^17^, ^18^ most likely reflecting the advanced disease status of the patients included in the REDUCER-1 study. However, given the limited sample size of the AngioNOCAD group, generalization of the MACE rate from this subanalysis is exploratory and requires additional confirmation.

### Study limitations

The population with AngioNOCAD studied in the current report should be considered distinct from the patients conventionally considered to have ANOCA who do not have the same history of extensive prior revascularization and frequently tend to be younger with a greater proportion of women.^1^^,^^34^^,^^35^ It is important to note the definition of AngioNOCAD in this study was <70% stenosis, although similar to previous reports,^16^, ^17^, ^18^ is different to prior studies which have used a threshold of <50% stenosis as a definition.^13^^,^^36^ In addition, the registry had no control arm and a placebo effect^4^^,^^5^ cannot be excluded to account in part for the observed changes in symptoms in the 2 groups. The study was also not sufficiently powered for this sub analysis of differences in treatment response between the AngioNOCAD and obstructive CAD group and should only be considered hypothesis generating. The registry was also limited in its lack of formal evaluation of epicardial and microvascular physiology by coronary function testing. Randomized controlled trials examining the efficacy of the CS Reducer in patients both with RA and myocardial ischemia in the setting of obstructive CAD COSIRA -II, NCT05102019) and nonobstructive CAD with confirmed CMD (REMEDY-PILOT, NCT05492110, and REDUCE CMD, NCT06898541) are ongoing.

## Conclusions

This subanalysis of the REDUCER-I registry demonstrates that CS Reducer implantation in patients with RA and AngioNOCAD resulted in significant reduction in anginal symptoms and improved quality of life through 1 year (Central Illustration). The symptomatic benefits in the AngioNOCAD group were similar to those seen in patients with obstructive CAD. These results, although consistent with preliminary reports of the potential beneficial effects of CS Reducer in patients with ANOCA, can only be considered hypothesis-generating and will require confirmation by ongoing randomized double-blind sham controlled clinical trials.

**Central Illustration:**
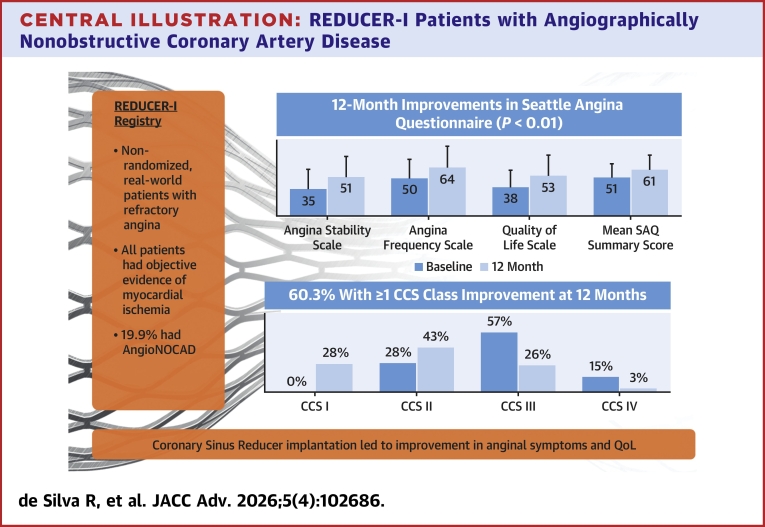
**REDUCER-I Patients with Angiographically Nonobstructive Coronary Artery Disease** In the REDUCER-I registry, patients with RA and angiographically nonobstructive CAD had significant improvements in their symptoms and quality of life suggesting that CS Reducer implantation may be an effective therapy in these patients. AngioNOCAD = angiographically nonobstructive coronary artery disease; CCS = Canadian Cardiovascular Society; QoL = quality of life; SAQ = Seattle Angina Questionnaire.

## Funding support and author disclosures

The REDUCER-I study is sponsored by Shockwave Medical Inc, Santa Clara, CA, USA, although no specific funding was provided for this analysis. Dr de Silva serves as a proctor for and receives honoraria from Shockwave Medical Inc. Dr Banai has received honoraria and is on the advisory board for Shockwave Medical Inc. Drs Luyapan and West are employees of Shockwave Medical Inc. Dr Verheye serves as a proctor for and has received honoraria from Shockwave Medical Inc. All other authors have reported that they have no relationships relevant to the contents of this paper to disclose.
