# Supplementary material for: Coronary Sinus Reduction for REDUCER-I Patients With Refractory Angina and Angiographically Nonobstructive Coronary Artery Disease
Source: JACC Adv. 2026 Mar 20;5(4):102686. doi: 10.1016/j.jacadv.2026.102686 (PMC13019784; doi:10.1016/j.jacadv.2026.102686)
Supplement: Supplemental_Material [file mmc1.docx]

**SUPPLEMENTAL APPENDIX**

The following table includes the CCS classes of the obstructive CAD and AngioNOCAD groups at baseline and 12 months, along with the p-value comparing the distributions of the two groups. There were no statistically significant differences in the distributions of the CCS classes in the two groups.

| **Measure** | **Obstructive CAD**  **% (n/N)** | **ANOCA**  **% (n/N)** | **P-value** |
| --- | --- | --- | --- |
| **Baseline CCS Assessment** |  |  | 0.13 |
| Grade I | 0.0% (0/245) | 0.0% (0/61) |  |
| Grade II | 29.8% (73/245) | 27.9% (17/61) |  |
| Grade III | 63.7% (156/245) | 57.4% (35/61) |  |
| Grade IV | 6.5% (16/245) | 14.8% (9/61) |  |
| **1 Year CCS Assessment** |  |  | 0.12 |
| Grade I | 40.3% (91/226) | 27.6% (16/58) |  |
| Grade II | 42.5% (96/226) | 43.1% (25/58) |  |
| Grade III | 15.0% (34/226) | 25.9% (15/58) |  |
| Grade IV | 2.2% (5/226) | 3.4% (2/58) |  |

The following table shows the change in CCS class for patients in the obstructive CAD and AngioNOCAD groups. There were no statistically significant differences in the patients who had ≥2, ≥1, no change, or <1 change in CCS class between the two groups.

| **Change in CCS at 1 Year from Baseline** | **Obstructive CAD**  **% (n/N)** | **AngioNOCAD**  **% (n/N)** | **P-value** |
| --- | --- | --- | --- |
| Improved by >= 2 Classes | 26.1% (59/226) | 17.2% (10/58) | 0.17 |
| Improved by >= 1 Class | 71.7% (162/226) | 60.3% (35/58) | 0.11 |
| No Change | 25.2% (57/226) | 37.9% (22/58) | 0.07 |
| Worsened by 1 Class | 2.7% (6/226) | 1.7% (1/58) | 1.00 |

SAQ scores for Obstructive CAD and AngioNOCAD patients at baseline and 12 months from Figure 4 are shown below. All scores were significantly improved from baseline for the obstructive CAD group, and except for physical limitation scale and treatment satisfaction scale, all scores were also significantly improved for the AngioNOCAD group. Between the two groups, the obstructive CAD group had higher SAQ scores in most domains than the AngioNOCAD group although this study was not powered for between-group comparisons.

|  | **Baseline** | | **12 Month** | | |
| --- | --- | --- | --- | --- | --- |
| **SAQ Subscale Measures** | **Obstructive CAD** | **ANOCA** | **Obstructive CAD** | **ANOCA** | **P-value***^1^* |
| **Physical Limitation Scale** |  |  |  |  |  |
| Mean ± StdDev (N) | 54.5 ± 24.9 (195) | 53.5 ± 24.0 (52) | 68.2 ± 26.5 (195) | 56.9 ± 25.6 (52) | 0.004 |
| Change from Baseline P-value*^2^* |  |  | <.0001 | 0.15 |  |
| **Angina Stability Scale** |  |  |  |  |  |
| Mean ± StdDev (N) | 42.2 ± 24.9 (222) | 36.0 ± 25.9 (57) | 55.0 ± 23.4 (222) | 51.3 ± 27.7 (57) | 0.63 |
| Change from Baseline P-value*^2^* |  |  | <.0001 | 0.003 |  |
| **Angina Frequency Scale** |  |  |  |  |  |
| Mean ± StdDev (N) | 51.7 ± 27.2 (222) | 49.6 ± 25.7 (57) | 71.7 ± 27.8 (222) | 63.7 ± 28.6 (57) | 0.15 |
| Change from Baseline P-value*^2^* |  |  | <.0001 | 0.0003 |  |
| **Treatment Satisfaction Scale** |  |  |  |  |  |
| Mean ± StdDev (N) | 79.8 ± 18.0 (222) | 78.3 ± 19.0 (57) | 85.8 ± 18.2 (222) | 78.8 ± 23.1 (57) | 0.06 |
| Change from Baseline P-value*^2^* |  |  | <.0001 | 0.82 |  |
| **Quality of Life Scale** |  |  |  |  |  |
| Mean ± StdDev (N) | 38.0 ± 23.6 (222) | 38.6 ± 23.1 (57) | 64.9 ± 26.1 (222) | 52.9 ± 27.7 (57) | 0.003 |
| Change from Baseline P-value*^2^* |  |  | <.0001 | 0.0004 |  |
| **Mean SAQ Summary Score***^3^* |  |  |  |  |  |
| Mean ± StdDev (N) | 47.8 ± 20.2 (222) | 47.3 ± 20.3 (57) | 69.1 ± 17.2 (222) | 60.8 ± 19.9 (57) | 0.001 |
| Change from Baseline P-value*^2^* |  |  | <.0001 | 0.0002 |  |
| *^1^*P-values were calculated using T-tests for differences in change from baseline between obstructive CAD and non-obstructive (ANOCA) CAD. *^2^*P-values were calculated using paired T-tests. *^3^*The SAQ summary score is based on the physical limitation, anginal frequency and quality of life scores. | | | | | |
